# Depressive Symptoms in a Patient with VEXAS Syndrome and Its Relationship with Depression: A Case Study

**DOI:** 10.1192/j.eurpsy.2025.2027

**Published:** 2025-08-26

**Authors:** Á. Esquembre García, M. D. L. E. Sánchez Escalonilla-Relea, P. Aguirre, M. Viaña Pérez, C. Herranz Serfaty, A. Muñoz San José, F. S. Mir Biribay, M. F. Bravo Ortiz

**Affiliations:** 1 Psychiatry, Hospital Universitario La Paz, Madrid, Spain

## Abstract

**Introduction:**

VEXAS syndrome is a newly recognized multisystem inflammatory disorder characterized by recurrent fevers, skin manifestations, and systemic symptoms, often leading to significant morbidity. While the physical aspects of this syndrome are increasingly documented, the psychiatric implications, particularly depressive symptoms, are less explored. This case study aims to elucidate depressive symptoms in a patient diagnosed with VEXAS syndrome, examine how these symptoms relate to prolonged diagnostic uncertainty, and assess the impact of receiving a definitive diagnosis on the patient’s mental health.

**Objectives:**

To evaluate the presence and severity of depressive symptoms in a patient with VEXAS syndrome. To analyze the psychological impact of prolonged diagnostic uncertainty on the patient’s mood. To investigate the effect of receiving a definitive diagnosis and a comprehensive treatment plan on the patient’s emotional well-being.

**Methods:**

This case report describes a 61-year-old male patient with VEXAS syndrome, admitted for further evaluation of his condition. He presented to the psychiatry service with complaints of low mood and morning asthenia. A thorough psychiatric assessment revealed a history of psychiatric hospitalization 30 years prior and ongoing treatment for an adjustment disorder since 2007. The assessment utilized standardized scales to measure depressive symptoms and documented the patient’s emotional state and coping mechanisms throughout his medical journey.

**Results:**

The patient experienced persistent low mood episodes since the onset of organic symptoms in 2019, exacerbated by multiple misdiagnoses and inadequate treatments. After receiving a diagnosis of VEXAS syndrome in July 2023, he reported significant improvements in mood and a reduction in suicidal ideation. He attributed these changes primarily to the clarity provided by the diagnosis and the development of a new treatment plan, rather than solely to adjustments in his antidepressant medication (sertraline, 100 mg). Although he tolerated the medication well, he emphasized that the sense of being understood significantly enhanced his motivation. Additionally, the patient reported vivid nightmares over the last two weeks but denied current suicidal thoughts.

**Conclusions:**

This case highlights the complex relationship between prolonged diagnostic uncertainty and depressive symptoms in chronic inflammatory diseases like VEXAS syndrome. The findings suggest that a definitive diagnosis and clear treatment strategy are crucial for improving mental health and overall well-being. This underscores the importance of a multidisciplinary approach that prioritizes both physical and psychological needs, enhancing the quality of care for patients navigating such complex conditions.

**Disclosure of Interest:**

None Declared

